# A case of gastric adenocarcinoma with pyloric gland-type infiltrating submucosa

**DOI:** 10.1186/s40792-024-01835-8

**Published:** 2024-04-07

**Authors:** Kaiho Hirata, Shusuke Yagi, Hideki Miyazaki, Kazuhiko Yamada, Naoki Akazawa, Naoki Enomoto, Kyoko Nohara, Chizu Yokoi, Toru Igari, Norihiro Kokudo

**Affiliations:** 1 Department of Surgery, Center Hospital of the National Center for Global Health and Medicine, 1-21-1, Toyama, Shinjuku, Tokyo, 162-8655 Japan; 2 Department of Pathology, Center Hospital of the National Center for Global Health and Medicine, 1-21-1, Toyama, Shinjuku, Tokyo, 162-8655 Japan; 3 Department of Gastroenterology, Center Hospital of the National Center for Global Health and Medicine, 1-21-1, Toyama, Shinjuku, Tokyo, 162-8655 Japan

**Keywords:** Immunohistochemical staining, Gastric cancer, Gastric adenocarcinoma with pyloric gland type

## Abstract

**Background:**

The development of immunohistochemical staining has revealed that gastric adenocarcinoma with the gastric phenotype can be divided into the foveolar, fundic gland, and pyloric gland phenotypes. Gastric adenocarcinoma of the pyloric gland type is difficult to diagnose using biopsy because of its low atypia and rarity. Herein, we describe a case of gastric adenocarcinoma of the pyloric gland type that was diagnosed immunohistochemically after endoscopic resection.

**Case presentation:**

A 67-year-old man was referred to our hospital for the diagnosis and treatment of a 30-mm elevated lesion on the lesser curvature side of the middle of the gastric body. Although four biopsies were performed, it was difficult to determine whether the lesion was benign or malignant. Therefore, endoscopic submucosal dissection was performed, and the presence of tumor cells infiltrating the submucosa with venous invasions was identified. Immunohistochemical staining revealed that the tumor cells were positive for MUC5AC and MUC6 and negative for Pepsinogen I and H + /K + -ATPase. From the above findings, he was diagnosed as having gastric adenocarcinoma with pyloric gland type. The patient underwent a laparoscopic distal gastrectomy and was discharged without any adverse events.

**Conclusions:**

Gastric adenocarcinoma of the pyloric gland type is a rare disease, and endoscopic resection can serve as a viable diagnostic option for this condition when it is difficult to diagnose using biopsy. Immunohistochemical pathology images can aid in the diagnosis of gastric adenocarcinoma of the pyloric gland type.

## Background

Recently, the development of immunohistochemical staining has revealed that gastric adenocarcinoma differentiates into the gastric and intestinal phenotypes. The gastric phenotype is further divided into the foveolar, fundic gland, and pyloric gland phenotypes [1]. Gastric adenocarcinoma of the fundic gland type is a subtype of gastric adenocarcinoma that was proposed in 2010 [2] and newly included in the WHO classification in 2019 [3]. However, gastric adenocarcinomas with gastric phenotypes other than gastric adenocarcinoma with fundic gland type have not yet been widely recognized. Each subtype of gastric adenocarcinoma with gastric phenotype is characterized by low atypia and is often difficult to diagnose [4–6]. Therefore, as with other low-grade well-differentiated adenocarcinomas of the gastric phenotype (LG-WDA-G), gastric adenocarcinoma of the pyloric gland type is difficult to diagnose on biopsy specimens because of its low atypia and rarity. So far, only one case of gastric adenocarcinoma of the pyloric gland type has been reported. In this report, we describe a case of gastric adenocarcinoma of the pyloric gland type which was diagnosed immunohistochemically after endoscopic resection due to diagnostic difficulties on biopsy specimens. In this report, detailed immunohistochemical staining was performed, and the immunohistological pathology images in this report will be helpful in the diagnosis of gastric adenocarcinoma of the pyloric gland type.

## Case presentation

A 67-year-old man visited a local hospital with abdominal pain. He underwent esophagogastroduodenoscopy (EGD), which revealed a 30 mm-sized elevated lesion with a concave surface and mucus production on the lesser curvature side of the middle of gastric body; however, the biopsy specimens obtained showed no malignant findings. The patient underwent another EGD the following month, and the biopsy specimens revealed pyloric adenoma (Group 3) with no malignant findings. The HE-stained specimen was positive for *Helicobacter pylori* infection, and he underwent the required eradication therapy.

**Fig. 1:**
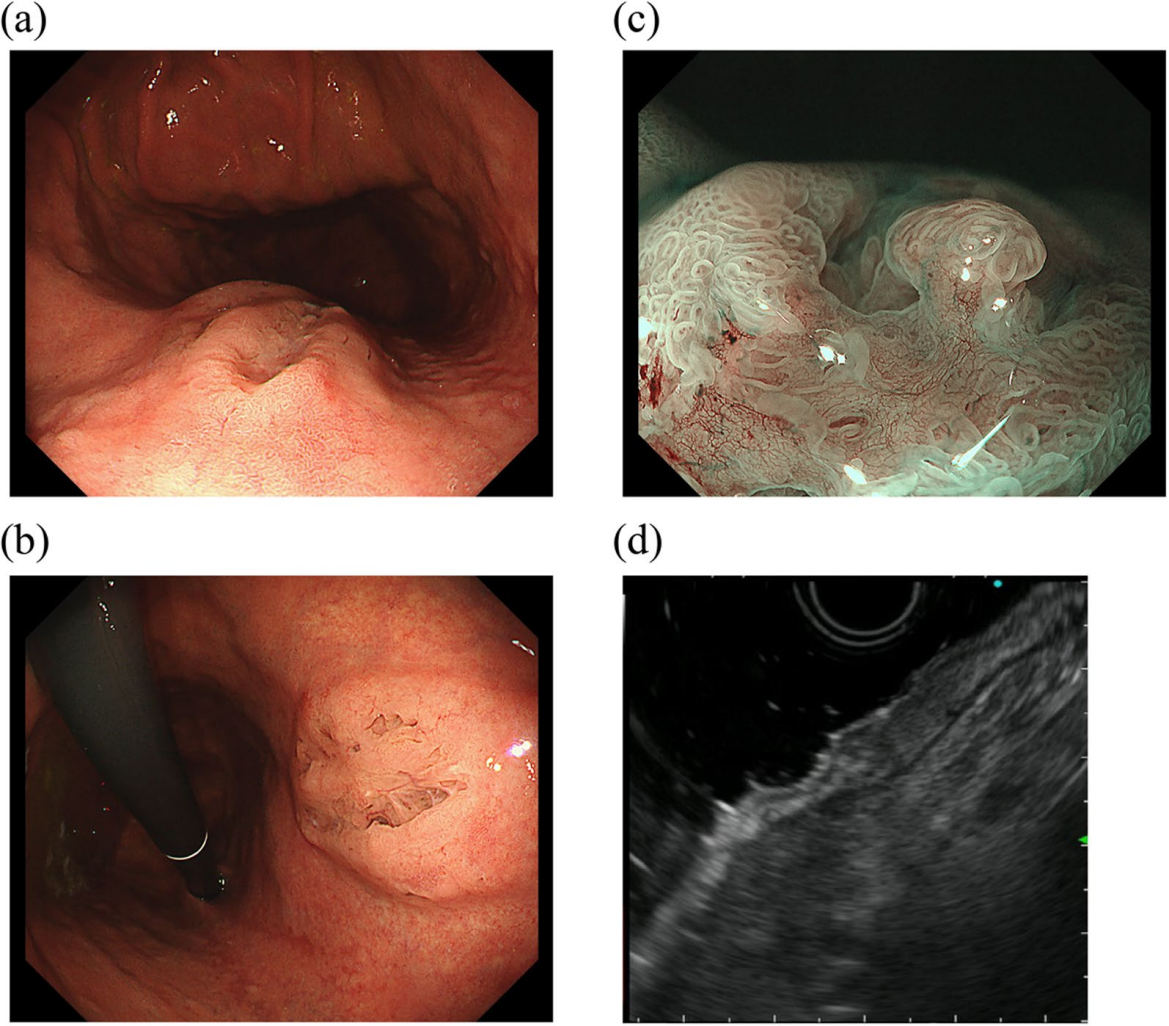
0–IIa + IIc lesion measuring 30 mm in size with a depressed surface at the lesser curvature side of the middle of the gastric body (**a** and **b**). Magnifying NBI revealed the vascular structure at the center of the lesion (**c**). Endoscopic ultrasonography revealed an isoechoic tumor with an elongated anechoic area suggestive of an ectopic pancreas, and also showed that the deep third layer and forth layer were remained (**d**)

**Fig. 2:**
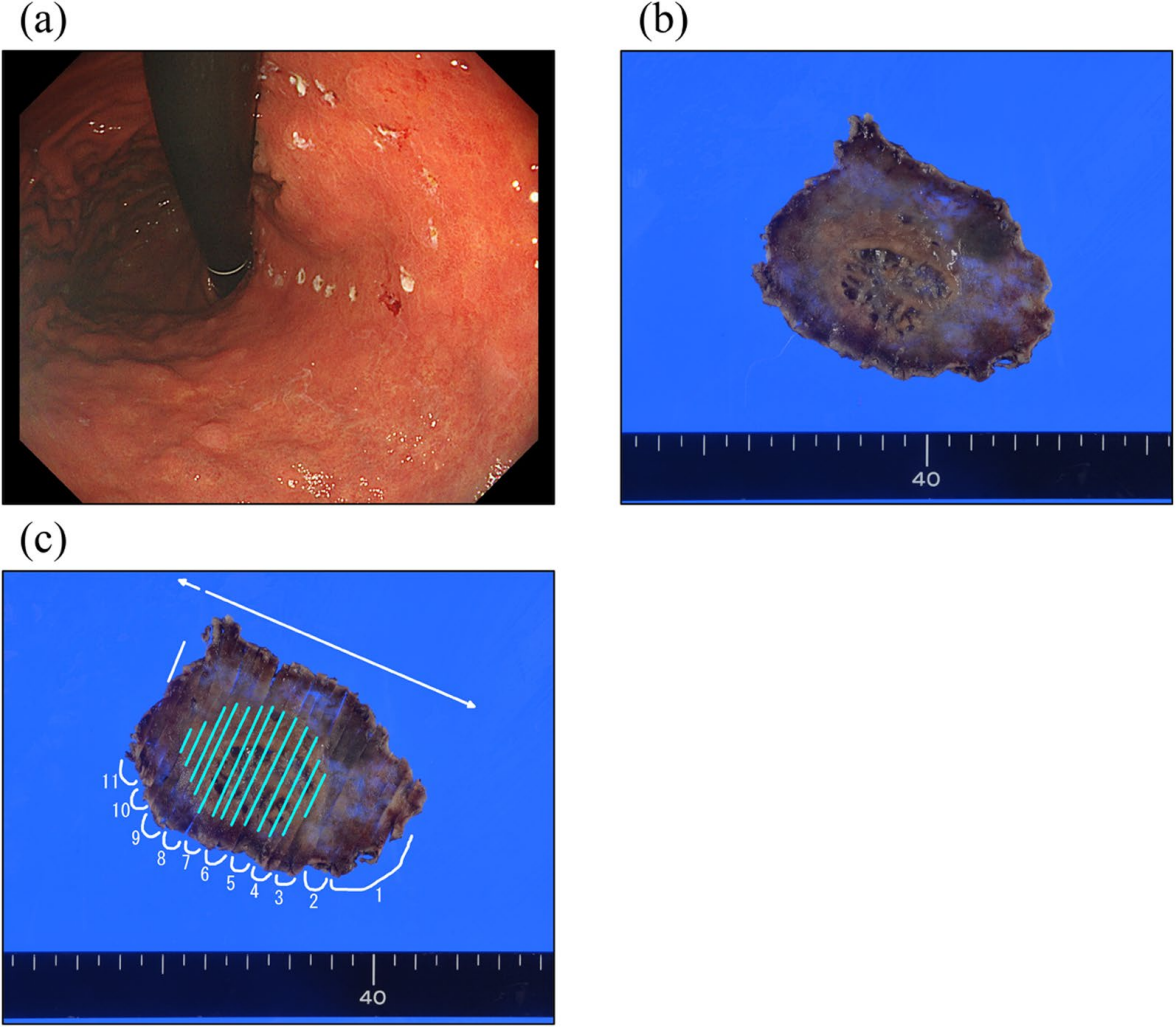
Gross findings of the resected specimens by endoscopic submucosal dissection, measuring 68 × 50 mm in size, with a 39 × 32-mm SMT-like elevation, a 26 × 17-mm ulcer was observed on the side of the lesion (**a**–**c**)

**Fig. 3:**
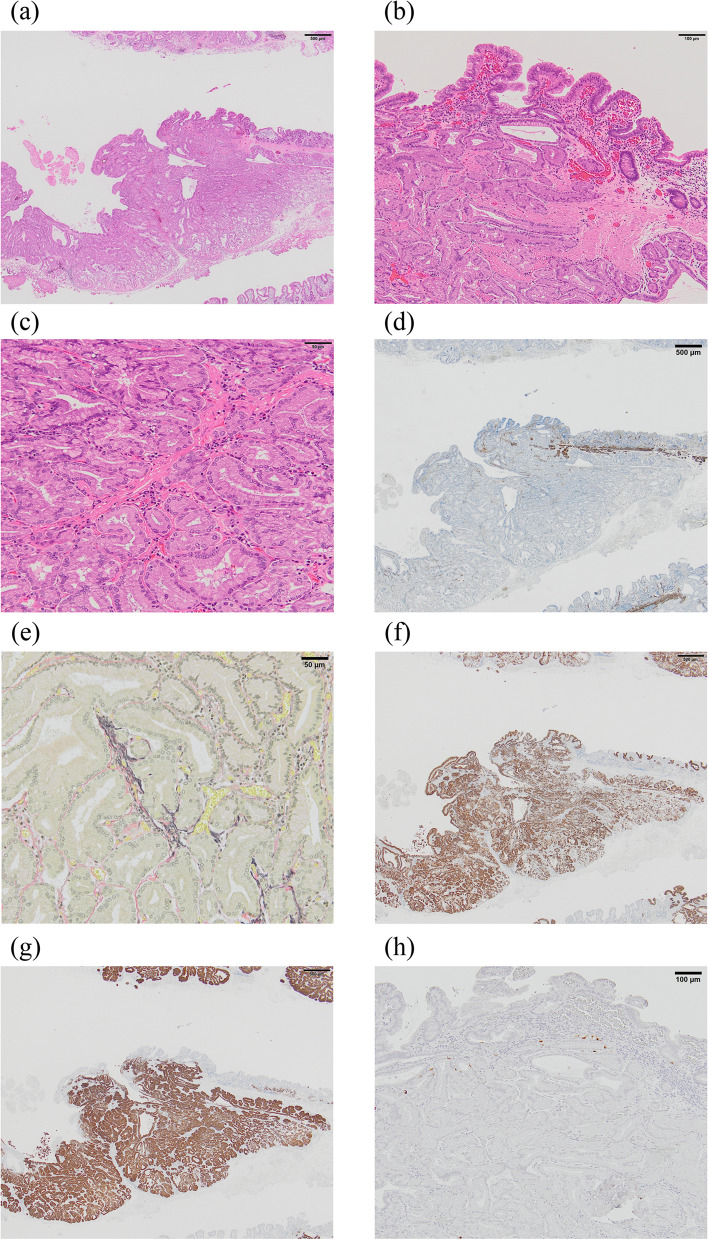

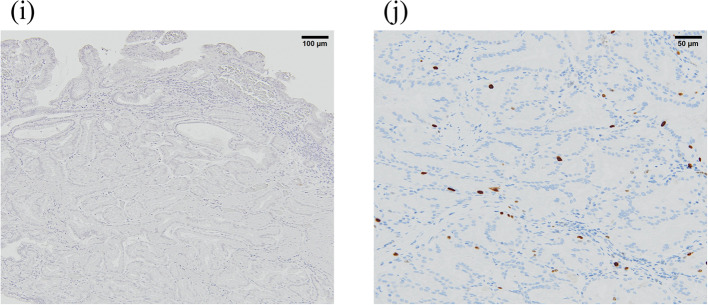
Histopathological findings of the tumor. **a**–**c** Lobular proliferation of pyloric gland-like glands was observed. **d** Tumor glands infiltrated submucosal tissues. **e** Venous invasion was observed in Elastica van Gierson staining. **f**–**i** Immunohistochemical staining of MUC5AC (**f**) and MUC6 (**g**), Pepsinogen-I (**h**), and H + /K + -ATPase. (**j**) The Ki-67 labeling index of the tumor is approximately 5%

Three months later, an EGD was restudied and biopsies were performed again; however, no malignant findings were observed. Then, the patient was referred to our hospital for a thorough examination of the lesion. An EGD revealed a 30-mm 0–IIa + IIc lesion with a depressed surface on the lesser curvature side of the middle of the gastric body (Fig. 1a, b). Magnifying narrow band imaging (NBI) revealed that the center of the lesion was white in color and vascularized (Fig. 1c). Endoscopic findings showed the good wall extensivity of the tumor, but the wall thickening remained by air suppression. Biopsy specimens revealed the presence of atypical cells and proliferating glandular ducts with irregular branching and fusion, raising suspicion of pyloric gland adenoma or pyloric gland adenocarcinoma. Endoscopic ultrasonography (EUS) showed isoechoic tumor with an elongated anechoic area, suggesting an ectopic pancreas. In addition, EUS findings showed the deep third layer and forth layer were remained, suggesting that the isoechoic tumor did not invade the deep submucosa and muscularis propria. Since it was hard to determine whether the lesion was benign or malignant, endoscopic submucosal dissection was performed. The resected specimen measured 68 × 50 mm. A 39 × 32-mm submucosal tumor (SMT)-like elevation was found, forming an ulcer measuring 26 × 17-mm (Fig. 2). Histopathologically, the lobular proliferation of pyloric grand-like acinar structures was observed but there were neither chief cells nor parietal cells. These atypical glands infiltrated the submucosal tissue (Fig. 3a–d). Venous invasion was observed (Fig. 3e). Immunohistochemically, tumor cells were positive for both MUC5AC and MUC6 (Fig. 3f and g), while pepsinogen-I and H^+^/K^+^-ATPase were both negative (Fig. 3h, i). The tumor’s Ki-67 labeling index was approximately 5% within the tumor (Fig. 3j). Contrast-enhanced computed tomography revealed no obvious lymph node or distant metastases. Based on the above, the patient was diagnosed with gastric adenocarcinoma of the pyloric gland type, pType 0-IIa + IIc, 39 × 32 mm, pT1b2(SM2) (2200 µm), UL0, Ly0, V1, pHM0, pVMX, cN0M0 cStage I. Due to submucosal layer infiltration and venous invasion, a laparoscopic distal gastrectomy with D1 + lymph node dissection was performed three months after the endoscopic submucosal dissection. The pathological findings of the resected specimen showed the absence of a residual tumor and no lymph node metastasis in 42 lymph nodes that were dissected. On postoperative day 3, the patient started oral intake, and he was discharged on postoperative day 7.

## Discussion

LG-WDA-G has three subgroups, which are gastric adenocarcinoma of the foveolar, fundic gland, and pyloric gland types [1]. The fundic gland type is further divided into gastric adenocarcinoma of the fundic gland type (GAFG) and gastric adenocarcinoma of the fundic gland mucosa type (GAFGM) [7]. In this case, in addition to mucin histochemistry, the diagnosis of gastric adenocarcinoma of the pyloric gland type was made using immunohistochemistry staining for H^+^/K^+^-ATPase, a staining marker of gastric parietal cells, and pepsinogen-I, a staining marker of chief cells and mucous neck cells. Immunohistochemistry is useful for distinguishing the three subgroups from each other, and the characteristic immunohistochemical staining findings are summarized in Table 1.

**Table 1:** Findings of each of the subtypes of low-grade well-differentiated adenocarcinoma of gastric phenotype on immunohistochemical staining

	MUC2	CD10	MUC5AC	MUC6	Pepsinogen-I	H^+^/K^+^-ATPase
A. Foveolar type	−	−	+	−	−	−
B. Fundic gland type						
a. GAFG*	−	−	−	+	+	+
b. GAFGM**	−	−	+	+	+	+
C. Pyloric gland type	−	−	+	+	−	−

GAFG* gastric adenocarcinoma of the fundic gland type, GAFGM** gastric adenocarcinoma of the fundic gland mucosa type

Pyloric metaplasia is a metaplastic change of mucous neck cells in fundic glands caused by *H. pylori* infection, and it is considered a regenerative change that serves to compensate for damage to the gastric mucosa [8]. Pyloric metaplasia occurs by replacing mucin-rich oxyntic glands secreted by mucous neck cells of pyloric-type glands, which is positive for MUC6 [9]. Pyloric adenomas, despite exhibiting the pyloric gland phenotype, mostly arise from the fundic gland area with chronic gastritis or autoimmune gastritis [10, 11]. Similarly, the fact that in both cases of the gastric adenocarcinoma of the pyloric gland type (including the present case), the tumor cells originate from the fundic gland area, and the presence of gastritis in the background mucosa suggests that pyloric gland metaplasia may play an important role in the histogenesis of gastric adenocarcinoma of the pyloric gland type. In this case, biopsies were performed four times; however, the diagnosis was not made because the tumor cells were well differentiated and had low atypia, which is one of the pathological characteristics of LG-WDA-G. It was reported that LG-WDA-G, even with repeated biopsies, could not differentiate LG-WDA-G from inflamed or regenerative changes with hematoxylin–eosin staining alone [12]. Thus, sufficiently deep tissue sampling (for example, endoscopic resection) is critical to making the correct diagnosis. In addition, it is known that LG-WDA-G tends to have an undermining growth pattern [13]. This indicates that LG-WDA-G tends to infiltrate structures deeper than muscularis mucosa, which makes it more likely for the patient to have distant and lymphatic metastases. As with the study reported by Mochizuki, which shows a patient with advanced gastric adenocarcinoma of the pyloric gland type (pT3N3a), our case showed submucosal infiltration and venous invasion [1]. Therefore, an accurate diagnosis is necessary.

## Conclusions

Gastric adenocarcinoma of the pyloric gland type is a rare disease, and endoscopic resection could be a viable diagnostic option when it is difficult to make a biopsy-based diagnosis. Although its rate of atypia is low, cases of venous invasion and advanced carcinoma suggest the possibility of high malignancy. Further case series and investigations are needed to clarify the clinicopathological features of gastric adenocarcinoma of the pyloric gland type. Our case of gastric adenocarcinoma of the pyloric gland type demonstrated that this entity can be underrecognized or misdiagnosed as benign tumor if the clinicians are not aware of this entity.

## Data Availability

All data on which the conclusions of this case report are based are included in the present publication.

## Abbreviations

LG-WDA-G: Low-grade well-differentiated adenocarcinoma of gastric phenotype
EGD: Esophagogastroduodenoscopy
NBI: Narrow band imaging
EUS: Endoscopic ultrasonography
SMT: Submucosal tumor
GAFG: Gastric adenocarcinoma of the fundic gland type
GAFGM: Gastric adenocarcinoma of the fundic gland mucosa type
